# Distinct effects of citric acid on primary and anchor roots

**DOI:** 10.1093/plphys/kiag309

**Published:** 2026-05-23

**Authors:** Arijit Mukherjee

**Affiliations:** Howard Hughes Medical Institute and Department of Biology, University of North Carolina at Chapel Hill, Chapel Hill, NC 27599-3280, United States

Citric acid (CA) is a vital organic acid produced and naturally secreted at high concentrations around roots in some plants (Tahjib-Ul-Arif et al 2021). The exuded CA acidifies the immediate region surrounding roots, enhancing the bioavailability of essential nutrients like phosphorus and iron (Du et al 2024). Inside plant tissues, CA is integral for maintaining cellular energetics and transporting iron through the vasculature by forming a complex with iron. Consequently, CA treatment on plants holds agricultural relevance for mitigating heat, drought, salinity, and heavy metal stresses (Gao et al 2010; Tahjib-Ul-Arif et al 2021).

The Arabidopsis root is composed of a primary root (PR), lateral roots protruding from the PR, and anchor roots (ARs) emerging at the root–hypocotyl junction. This root architecture is highly dynamic, allowing plants to respond to fluctuating environmental signals. Exogenous CA treatment was shown to inhibit primary root growth but promote the formation of ARs in Arabidopsis (Zhang et al 2023). In this issue of *Plant Physiology*, the same group has provided the mechanistic basis for such observation, highlighting the tissue-specific role of CA in root development.

CA treatments in in vitro plant growth systems may substantially reduce pH with pleiotropic effects on plant development, obscuring the study of the specific effects of CA. To mitigate this, the authors used a sodium citrate–citric acid buffered growth medium, allowing them to study dose-dependent effects of CA on Arabidopsis roots at physiologically relevant concentrations. Increasing the exogenous CA (1–5 mM) significantly reduced PR length, whereas the same concentrations significantly increased the number of ARs. This established opposing developmental effects of CA on primary and anchor roots. Contrary to the expected role of CA in facilitating nutrient uptake, it did not enhance the nutrient levels in either root or shoot; rather, CA reduced certain nutrients in the shoot.

The authors next examined the transcriptional response of PRs and ARs in response to CA treatment via bulk RNA sequencing. Notably, most of the CA-regulated genes in PRs displayed opposite regulation in ARs, reflecting the contrasting developmental effects of CA in these root types. Gene Ontology term enrichment analysis of these genes revealed reactive oxygen species (ROS) homeostasis as a critical regulator for the observed phenotype. Analyses of hydrogen peroxide (H_2_O_2_)–specific fluorescent probe *35S-Hyper* in hypocotyl and mature root regions revealed that CA application has a more pronounced effect on mature roots than hypocotyls, suggesting that the spatial distribution of H_2_O_2_ might be implicated in AR development.

Further analysis of the RNA sequencing data revealed a strong impact of CA on genes implicated in converting ROS into stable compounds. Most of the Class III peroxidase genes that use H_2_O_2_ as an electron acceptor were upregulated in PRs in response to CA application, whereas these genes were downregulated in ARs. This was further validated via reverse transcription quantitative PCR and a series of peroxidase activity assays in roots, suggesting that CA reduces H_2_O_2_ by increasing peroxidase activity, specifically in primary roots. To connect CA-mediated H_2_O_2_ reduction and the observed phenotypes, the authors used known H_2_O_2_ scavengers to phenocopy CA-mediated effects on Arabidopsis roots. Indeed, application of these scavengers at concentrations comparable to the reduction of H_2_O_2_ levels upon CA treatment phenocopied the overall root architecture. The results established that altering H_2_O_2_ levels is sufficient to induce the observed effects on root architecture.

Given the role of Class III peroxidase in lignin polymerization, the authors further investigated if CA treatment could induce lignification. *Trans*–cinnamic acid (t-CNA), a precursor of lignin biosynthesis, could phenocopy CA effects (Herrero et al 2013). Although t-CNA treatment supplemented the effect of CA on roots, it was alone sufficient to induce AR formation. This observation was further supported by lignin biosynthesis mutants (*c4h*, *4cl1*, and *4cl3*), which exhibited increased resistance to the root growth inhibition caused by CA, t-CNA, or their combined treatment. Collectively, these findings identify lignin biosynthesis as a major mechanism underlying CA-induced AR formation.

Since H_2_O_2_ levels are well recognized for their role in the development of the quiescent center (QC) in roots, the authors also investigated whether CA treatment also influences this process (Zluhan-Martínez et al 2025). Interestingly, the CA-induced QC phenotype resembled *ERF115* overexpression Arabidopsis lines by enhancing the proliferation and disorganization of QC (Heyman et al 2013). Analyses of the GUS reporter line of ERF115 in response to CA treatment further confirmed its induction in response to CA treatment. Interestingly, this response was specific to CA and not to malic acid, a TCA intermediate. Therefore, the QC phenotypes are specifically and partially regulated via CA-induced *ERF115* overexpression.

In summary, Zhang et al (2023) describe how CA affects peroxidases, lignin synthesis, QC formation, and PR and AR growth (Fig. 1). A major finding presented in this study involved differential integration of the same exogenous signal in genetically identical tissue types. However, the communication between root and hypocotyl and its implications for AR development warrants further investigation. Since ARs help plants adapt toward environmental challenges and nutrient uptake, these findings hold potential agricultural applications for developing lodging-resistant crops.

**Figure 1:**
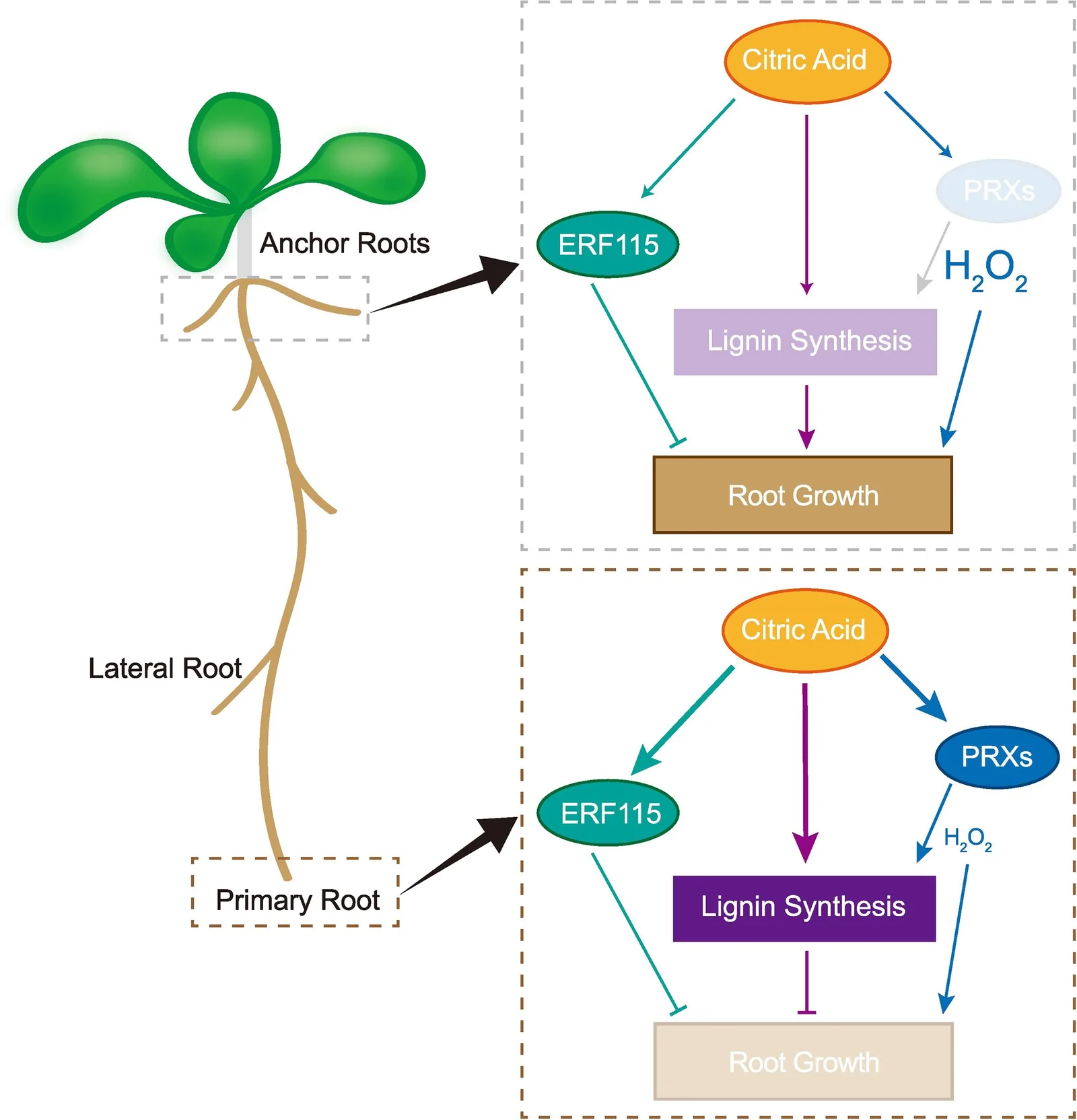
A schematic summarizing the proposed pathways through which CA differentially impacts anchor and primary roots. CA treatment induces differential effects on ERF115 expression, lignin synthesis, and Class III peroxidases (PRXs) in primary and anchor roots. Figure is taken from Zhang et al (2026).

**Recent related articles in *Plant Physiology*:**

- Kunkowska (2025) Rooting for survival: OsCDPK5 and OsCDPK13 are crucial for rice acclimation to low-oxygen conditions. Plant Physiology 197:kiae359.
- Cohen-Hoch et al (2025) Osmotic stress in roots drives lipoxygenase-dependent plastid remodeling through singlet oxygen production. Plant Physiology 197:kiae589.

## Data Availability

Not applicable.

## References

[kiag309-B1] Cohen-Hoch D et al 2025. Osmotic stress in roots drives lipoxygenase-dependent plastid remodeling through singlet oxygen production. Plant Physiol. 197:kiae589. 10.1093/plphys/kiae589. 39498840

[kiag309-B2] Du Z et al 2024. Recovery of iron phosphate from secondary sludge by ascorbic and citric acids. J Environ Chem Eng. 12:111917. 10.1016/j.jece.2024.111917.

[kiag309-B3] Gao Y et al 2010. Improvement of phytoextraction and antioxidative defense in *Solanum nigrum* L. under cadmium stress by application of cadmium-resistant strain and citric acid. J Hazard Mater. 181:771–777. 10.1016/j.jhazmat.2010.05.080. 20566243

[kiag309-B4] Herrero J, Esteban-Carrasco A, Zapata JM. 2013. Looking for Arabidopsis thaliana peroxidases involved in lignin biosynthesis. Plant Physiol Biochem. 67:77–86. 10.1016/j.plaphy.2013.02.019. 23545205

[kiag309-B5] Heyman J et al 2013. ERF115 controls root quiescent center cell division and stem cell replenishment. Science. 342:860–863. 10.1126/science.1240667. 24158907

[kiag309-B6] Kunkowska AB . 2025. Rooting for survival: OsCDPK5 and OsCDPK13 are crucial for rice acclimation to low-oxygen conditions. Plant Physiol. 197:kiae359. 10.1093/plphys/kiae359. PMC11663585 38935573

[kiag309-B7] Tahjib-Ul-Arif M et al 2021. Citric acid-mediated abiotic stress tolerance in plants. Int J Mol Sci. 22:7235. 10.3390/ijms22137235. 34281289 PMC8268203

[kiag309-B8] Zhang T et al 2023. Chemical imaging reveals diverse functions of tricarboxylic acid metabolites in root growth and development. Nat Commun. 14:2567. 10.1038/s41467-023-38150-z. 37142569 PMC10160030

[kiag309-B9] Zluhan-Martínez E et al 2025. The MADS-box gene XAANTAL1 participates in Arabidopsis thaliana primary root growth and columella stem cell patterns in response to ROS, via direct regulation of PEROXIDASE 28 and RETINOBLASTOMA-RELATED genes. J Exp Bot. 76:411–432. 10.1093/jxb/erae415. 39377268 PMC11714753

